# Allogeneic CAR-T, CAR-NK, and *in vivo* CAR-T for immune-mediated inflammatory diseases: comparative evidence, platform trade-offs, and translational challenges

**DOI:** 10.3389/fimmu.2026.1931185

**Published:** 2026-10-28

**Authors:** Zhenguo Liang, Mengyun Mao, Hui Xie, Yuhan Xing, Dongze Wu

**Affiliations:** 1 Department of Rheumatology and Immunology, The Fifth Affiliated Hospital, Sun Yat-Sen University, Zhuhai, Guangdong, China; 2 Department of Research and Development, Antengene Corporation, Shanghai, China; 3 School of Public Health (Shenzhen), Sun Yat-Sen University, Shenzhen, Guangdong, China; 4 Department of Rheumatology and Immunology, Sichuan Provincial People’s Hospital, University of Electronic Science and Technology of China, Chengdu, China

**Keywords:** allogeneic CAR-NK, allogeneic CAR-T, B-cell depletion, immune reconstitution, immune-mediated inflammatory diseases, *in vivo* CAR-T

## Abstract

Immune-mediated inflammatory diseases (IMIDs) remain treatment-dependent in many patients with severe, refractory, or organ-threatening disease. Deep B-cell depletion using chimeric antigen receptor (CAR)-based therapies may induce clinical improvement and measurable immune reconstitution, but conventional autologous CAR-T manufacturing limits speed, standardization, and access. This structured narrative review compares allogeneic CAR-T, allogeneic CAR-natural killer (CAR-NK), and *in vivo* CAR-T. Bibliographic databases, trial registries, conference repositories, reference lists, and traceable sponsor, media, and regulatory sources were searched through 3 September 2026. Allogeneic CAR-T has the broadest clinical evidence base, although cohorts are small and mostly early-phase. All CAR-NK products with reported IMID clinical data are allogeneic; available studies suggest transient kinetics, repeat-infusion feasibility, and encouraging short-term tolerability, but durability is uncertain. Peer-reviewed *in vivo* evidence now includes a five-patient non-integrating LNP/linear mRNA report and a 16-patient integrating lentiviral report. Both are early, uncontrolled studies and neither supports class-wide inference. Disease-specific outcomes, background treatment, follow-up, and assays differ markedly. Complete treatment-free remission was distinguished from glucocorticoid withdrawal, immunosuppressant reduction, and remission on permitted therapy. CAR-cell, transgene DNA, and RNA detection were not pooled, and peripheral blood B-cell depletion was not treated as proof of tissue depletion. Naive-dominant B-cell recovery and declining autoantibodies indicate immune remodeling, not restored tolerance. No indirect efficacy ranking is justified. Prospective studies need standardized disease endpoints, treatment-free remission definitions, analyte-specific pharmacokinetics, paired blood–tissue pharmacodynamics, and long-term genomic, immune, and safety surveillance.

## 1 Introduction

Immune-mediated inflammatory diseases comprise heterogeneous disorders affecting multiple organs. Biologics and targeted small molecules have improved disease control, yet some patients remain dependent on chronic immunosuppression or develop refractory, relapsing, or organ-threatening disease (1–5). Goals are consequently expanding from short-term control toward sustained remission, organ protection, and, where possible, treatment withdrawal (6–10). Our previous review considered the broader transition from biologic and stem-cell therapies to targeted cellular therapies (11). The present review evaluates whether allogeneic CAR-T, allogeneic CAR-NK, and direct *in vivo* CAR engineering can address limitations of autologous manufacture without presuming superiority over established anti-CD20 therapy or off-the-shelf T-cell engagers (12–19).

Recent systematic and narrative reviews have synthesized CAR-T in systemic lupus erythematosus (SLE), refractory rheumatic autoimmune disease, or autoimmunity more broadly (20–22). Reviews have also addressed universal CAR engineering, CAR-NK biology, and *in vivo* CAR generation (13, 14, 18, 19, 23, 24). We add an IMID-specific cross-platform framework that links four questions: what clinical activity has been observed and from which source; how analyte-specific CAR kinetics relate to peripheral and tissue B-cell pharmacodynamics; whether medication withdrawal and immune reconstitution meet prespecified definitions; and how conditioning, safety, manufacturing, regulation, and implementation shape plausible roles. This is a comparative evidence map, not a head-to-head efficacy analysis. Clinical programs predominantly target CD19, B-cell maturation antigen (BCMA), or both, and concentrate on SLE/lupus nephritis, systemic sclerosis, inflammatory myopathies, and selected antibody-associated disorders. The conclusions therefore apply mainly to B-cell-associated systemic autoimmune disease, not to the full IMID spectrum.

## 2 Literature search, study selection, and evidence classification

### 2.1 Search strategy and eligibility

We conducted a structured narrative review rather than a registered systematic review. PubMed/MEDLINE, Embase, and Web of Science Core Collection were searched from inception through 3 September 2026. ClinicalTrials.gov, conference repositories, reference lists, major publisher feeds, and traceable sponsor, media, and regulatory sources were searched through the same date using platform, disease, product, target, sponsor, and trial-identifier terms. The overview of the search framework and source-specific adaptations are in the Supplementary Methods. Eligible peer-reviewed clinical reports identified by this unified cutoff were incorporated into the synthesis and descriptive counts; lower-provenance reports were retained only as explicitly labeled safety context and were not added to efficacy counts or evidence grades.

Eligible clinical sources reported original human data on allogeneic CAR-T, allogeneic CAR-NK, or *in vivo* CAR generation in IMIDs. Preclinical studies were included when they informed allo-evasion, delivery specificity, biodistribution, persistence, pharmacodynamics, or genomic risk. Oncology-only studies were excluded from the clinical synthesis but could provide clearly labeled engineering or safety context. Reviews were used for framing and citation tracking, not as primary clinical evidence.

### 2.2 Cohort consolidation and source status

For this review, a “cohort unit” denotes a consolidated set of one or more reports judged to describe a single, non-overlapping treated population; overlapping reports and updates are linked and counted once using the most complete report. Potential overlap was assessed using product, sponsor, institution, disease, enrollment period, dose, sample size, and follow-up. Peer-reviewed reports were prioritized over earlier abstracts for the same cohort; expanded updates were linked rather than double counted. Source status was recorded as peer-reviewed prospective study; peer-reviewed exploratory report; official conference report; traceable sponsor or media report; registry-only record; or preclinical evidence. These categories describe availability and provenance, not methodological quality. Interpretation separately considered design, sample size, completeness, follow-up, and overlap. No meta-analysis or indirect treatment comparison was performed.

Two non-conflicted authors independently reviewed each candidate record against prespecified eligibility criteria and recorded eligibility, cohort identity, overlap indicators, extracted data, and source status. Disagreements were resolved by joint re-review of the full text and relevant source record, followed by discussion and consensus. For possible overlap, product, sponsor, institution, disease, enrollment period, dose or schedule, sample size, follow-up, and trial identifiers were compared. Reports judged to represent the same treated population were linked as one cohort unit; unresolved relationships were flagged as uncertain. The industry-affiliated author provided technical clarification but did not make unilateral decisions.

Disease-specific endpoints and their assessment times were extracted without collapsing unlike outcomes into a common response rate. Medication outcomes were classified as complete treatment-free remission, sustained complete treatment-free remission, glucocorticoid-free remission, immunosuppressant-free remission, partial withdrawal or dose reduction, discontinuation without documented remission, continued background therapy, or inadequately reported status. Complete treatment-free remission required a documented disease-specific remission without systemic glucocorticoids, immunosuppressants, biologics, or other disease-modifying therapy; antimalarial use and duration were recorded separately. Discontinuation alone did not qualify.

CAR pharmacokinetic extraction recorded specimen, analyte (viable CAR-positive cells, transgene DNA, or RNA), assay, sampling window, and reported limit of detection or quantification. B-cell findings were separated into peripheral blood depletion/reconstitution and tissue or bone-marrow evidence. Absence of tissue sampling was not interpreted as absence of tissue disease.

## 3 Platform definitions and biological trade-offs

Allogeneic CAR-T denotes T cells derived from a donor or another non-autologous source and is not synonymous with universal CAR-T (25, 26). “Universal” describes an intended capacity for use across unrelated recipients, supported by product-specific strategies that reduce alloreactivity or rejection; it is a design objective, not proven compatibility with every recipient. TyU19 illustrates compatibility engineering: TRAC disruption reduces graft-versus-host reactivity, whereas HLA-directed modifications address host rejection (25–30).

“Off-the-shelf” describes manufacture and banking before patient-specific demand. FT819 combines an allogeneic iPSC source, TCR disruption, and clonal-bank manufacture, so the descriptors overlap within one product (27, 31–34). ADI-001, an allogeneic anti-CD20 gamma-delta CAR-T product listed in **Supplementary Table S2**, illustrates that off-the-shelf supply does not require a particular TCR/HLA-editing strategy. Cell source, compatibility design, and inventory availability should therefore be reported separately. Neither “universal” nor “off-the-shelf” guarantees conditioning-free treatment, absence of rejection or GVHD, or established long-term safety (12, 25–28, 35–37).

*In vivo* CAR-T is a family of vector–cargo combinations (13, 38, 39). Integrating viral vectors are intended to support durable expression but raise concerns about insertional genotoxicity, biodistribution, unintended transduction, shedding, and reversibility (40). A phase 1 report of JY231 now provides human proof of concept for T-cell-targeted integrating lentiviral delivery, but six-month vector-copy and integration-site observations do not establish durable functional CAR-T persistence or long-term genomic safety (41). Non-integrating viral or virus-like particle systems differ in tropism, episomal persistence, cargo, and redosing constraints (42). Targeted lipid nanoparticle (LNP)/linear mRNA systems generate transient expression without intended genomic integration; circular RNA may extend expression, whereas DNA or *in vivo* gene-editing systems can produce episomal or permanent changes (43). Undisclosed delivery or cargo should remain a separate category. **Figure 1**; **Table 1** summarize these distinctions.

**Figure 1:**
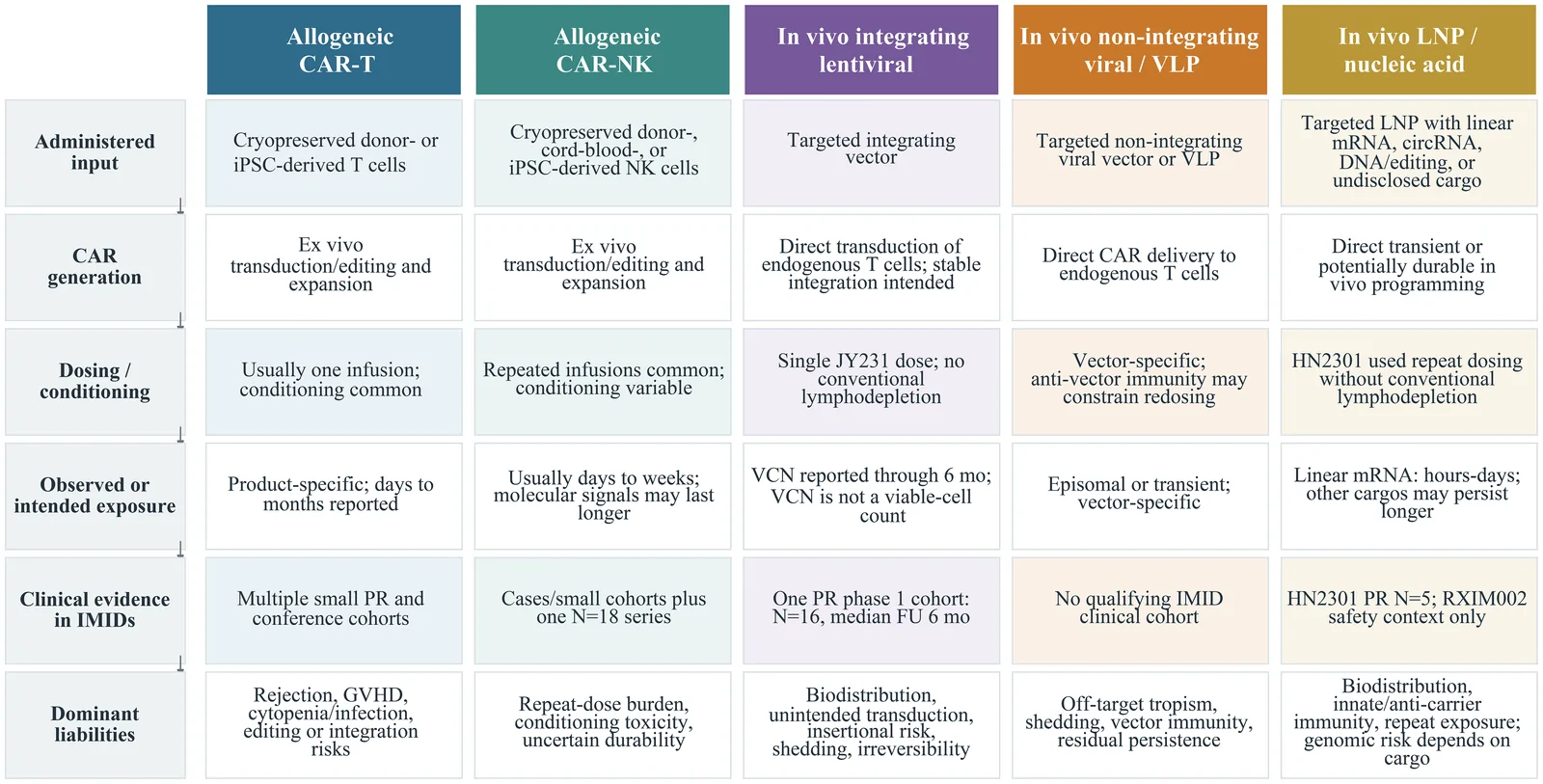
Platform lifecycles and platform-specific liabilities. The figure distinguishes administered allogeneic CAR-T and allogeneic CAR-NK products from *in vivo* integrating viral, non-integrating viral/virus-like particle, and nonviral nucleic-acid delivery systems. The integrating-viral column incorporates the early JY231 clinical report while preserving the distinction between reported vector copy number and viable-cell persistence and between six-month observations and long-term genomic safety. The nonviral category includes LNP/linear mRNA, LNP/circular RNA, DNA or *in vivo* editing, and undisclosed cargo. Operational characteristics are product- and modality-specific and should not be interpreted as established clinical roles. circRNA, circular RNA; GMP, good manufacturing practice; GVHD, graft-versus-host disease; IMID, immune-mediated inflammatory disease; iPSC, induced pluripotent stem cell; LNP, lipid nanoparticle; VCN, vector copy number; VLP, virus-like particle. Terminology: allogeneic, non-autologous cell source; universal, intended compatibility across unrelated recipients; off the shelf, premanufactured/banked supply. These descriptors may overlap but are not interchangeable.

**Table 1:** Platform-level comparison of allogeneic CAR-T, allogeneic CAR-NK, and *in vivo* CAR-T subtypes.

Domain	Allogeneic CAR-T	Allogeneic CAR-NK	*In vivo* integrating viral	*In vivo* non-integrating viral/VLP	*In vivo* LNP/nucleic acid
Administered product	Cryopreserved donor- or iPSC-derived T cells	Cryopreserved donor-, cord blood-, or iPSC-derived NK cells	Targeted integrating vector	Targeted non-integrating vector or VLP	Targeted LNP with linear mRNA, circRNA, DNA/editing cargo, or undisclosed cargo
CAR generation	Ex vivo transduction/editing and expansion	Ex vivo transduction/editing and expansion	Direct transduction of endogenous T cells with intended stable genomic integration	Direct delivery to endogenous T cells	Direct transient or potentially durable *in vivo* programming, depending on cargo
Observed/intended kinetics	Product-specific; days to months reported	Usually days to weeks; repeated infusion common	Durable expression intended; JY231 VCN reported through M6, but VCN is not a viable-cell count	Episomal or transient; vector-specific	Linear mRNA: hours–days; circRNA may persist longer; DNA/editing may be episomal or permanent
Conditioning	Common in current studies	Variable; often Flu/Cy	No conventional lymphodepletion in JY231; not established class-wide	Not established in IMIDs	No conventional lymphodepletion in HN2301; platform-specific otherwise
Principal acute risks	CRS, ICANS, GVHD, cytopenia, infection	CRS/infusion reaction, infection, conditioning toxicity	Grade 1 CRS and reversible neutropenia reported with JY231; inflammation and unintended transduction remain product-specific	Inflammation, tropism, vector immunity	CRS/innate activation, cytopenia, off-target uptake; formulation-specific
Principal delayed risks	Rejection, prolonged B-cell aplasia, hypogammaglobulinemia, editing/integration abnormalities	Repeat-dose burden and persistent immune dysfunction; long-term data sparse	Insertional genotoxicity, persistent off-target transduction, shedding, irreversibility; six-month data are not long-term safety	Residual persistence, shedding, immunity, redosing limits	Biodistribution, cargo/carrier persistence, anti-carrier immunity, repeat-exposure effects; genomic risk depends on cargo
Manufacturing bottleneck	Cell source, editing, expansion, cryorecovery, potency and comparability	Source/expansion, cytokine support, cryorecovery and multidose supply	GMP vector yield, targeting, integration profile and release	GMP vector/VLP yield, tropism, stability and immunity	LNP composition/stability, targeting, cargo integrity and lot consistency
Clinical evidence in IMIDs	Multiple small peer-reviewed and conference cohorts	Cases/small cohorts plus one 18-patient series	One peer-reviewed, single-arm 16-participant JY231 cohort; median follow-up 6 mo	No qualifying clinical cohort identified	One five-patient LNP/linear mRNA report; unadjudicated circRNA/LNP safety update only
Interpretation	Proof of concept; no established superiority	Proof of concept; durability uncertain	Early clinical proof of concept; efficacy, durability, redosing and long-term genomic safety unresolved	Preclinical/registry hypothesis	Modality-specific evidence only; no class-wide inference
Terminology notes	Allogeneic = non-autologous cell source; universal = intended compatibility across unrelated recipients; off-the-shelf = manufacture and banking before patient-specific demand. These descriptors may overlap but are not interchangeable.				

CAR, chimeric antigen receptor; circRNA, circular RNA; CRS, cytokine release syndrome; Flu/Cy, fludarabine/cyclophosphamide; GMP, good manufacturing practice; GVHD, graft-versus-host disease; ICANS, immune effector cell-associated neurotoxicity syndrome; IMID, immune-mediated inflammatory disease; iPSC, induced pluripotent stem cell; LNP, lipid nanoparticle; VLP, virus-like particle. Operational advantages remain hypotheses unless clinically demonstrated.

## 4 Comparative clinical activity and durability

### 4.1 Evidence landscape and comparability

The evidence is asymmetric. Consolidating overlapping updates yields approximately nine allogeneic CAR-T clinical cohort units (approximately 65 treated participants), five allogeneic CAR-NK units (36 participants), and two peer-reviewed *in vivo* cohort units: one non-integrating LNP/linear mRNA cohort (five participants) and one integrating lentiviral cohort (16 participants). These counts describe reported exposure, not pooled efficacy, and remain sensitive to incomplete overlap reporting. Registered programs without outcomes do not constitute clinical evidence. **Figure 2** maps cohort size, follow-up, and source status; **Table 2** supplies endpoint and assay context.

**Figure 2:**
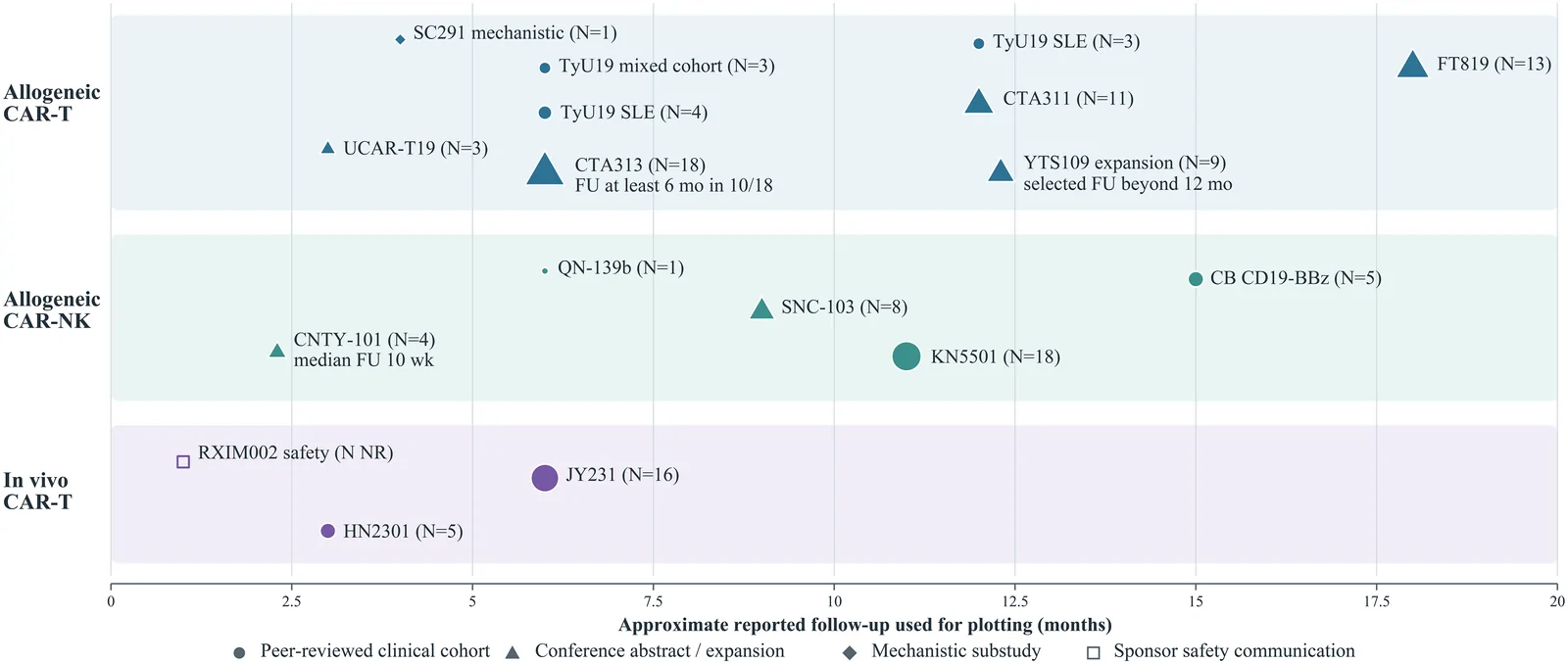
Clinical evidence landscape by cohort unit. Each plotted cohort unit represents a consolidated set of reports describing a single, non-overlapping treated population. Expanded updates replace overlapping earlier cohorts; marker area is proportional to reported N; position reflects approximate reported follow-up, not efficacy. The JY231 marker represents one peer-reviewed integrating-lentiviral cohort (N = 16; median follow-up 6 months). YTS109 is represented by its expanded N = 9 update, which supersedes the overlapping earlier N = 5 cohort. The plot maps available evidence and is not an efficacy analysis. FU, follow-up; NR, not reported.

**Table 2:** Representative clinical evidence for allogeneic CAR-T, allogeneic CAR-NK, and *in vivo* CAR-T in IMIDs.

Platform/product	Source status	Disease; N; follow-up	Disease-specific endpoint(s) and assessment	Clinical outcome	Medication status/complete TFR	CAR analyte, specimen, assay and last detection	Peripheral blood B-cell PD	Tissue/BM evidence	Safety/limitation
Allo CAR-T: TyU19	Peer-reviewed phase 1 (29)	SRP-IMNM and dcSSc; N = 3; 6 mo	Muscle enzymes/function; mRSS; organ imaging at 6 mo	Improvement in muscle, skin, lung and cardiac measures	Complete TFR not documented	Peripheral blood CAR+ cells; assay details in source; detected >3 mo	Depletion ~2 mo; recovery by 6 mo	Selected muscle/skin tissue and imaging assessments	No CRS/ICANS/GVHD reported; mixed diseases, N = 3
Allo CAR-T: TyU19	Two peer-reviewed cohorts (30, 44)	Refractory SLE; N = 4 and N = 3; 3–12 mo	SRI-4/SLEDAI and renal/serology assessments through 12 mo	Disease activity and proteinuria improved in small cohorts	Selected discontinuation reported; antimalarial status/duration incomplete, so complete sustained TFR not uniformly established	Peripheral blood CAR+ cells/transgene; product- and assay-specific; up to 12 mo in some	Depletion within days; recovery 3–12 mo	Selected renal histology/spatial profiling	Grade 1 CRS in one cohort; cytopenia, infection, Ig decline
Allo CAR-T: YTS109	Peer-reviewed phase 1 (45)	SLE/LN; N = 5; 6 mo	SRI-4 at M3; disease activity/renal outcomes through M6	5/5 SRI-4 at M3; one mild flare by M6	Medication discontinuation not sufficiently specified for complete TFR	Peripheral blood STAR-positive T cells; mostly undetectable by M2	Recovery from M2; naive-dominant	Selected renal tissue analyses	Grade 1 CRS 2/5; infection; short follow-up
Allo CAR-T: FT819	Official conferences (31–34, 58)	SLE/B-cell autoimmune disease; N = 13; ≤18 mo	SLE activity and renal endpoints at reported visits	Preliminary disease-activity/renal signals	3/7 stopped baseline glucocorticoids in one update; not complete TFR	Peripheral blood CAR signal; modality/source-specific; ~14 d	Conditioning associated with deeper depletion; reconstitution reported	Not systematically reported	No CRS >G2/ICANS/GVHD reported; evolving overlap and background-therapy confounding
Allo CAR-T: CTA311/CTA313	Conference plus sponsor-linked reports (46–49)	SLE/LN/AAV; N = 11 and N = 18; ≤12 mo	SRI-4, LLDAS, DORIS and organ endpoints at variable times	Preliminary outcome signals; denominators vary	Reported withdrawal/tapering lacks uniform drug lists, remission criteria or duration; complete TFR not established	Peripheral blood CAR+ cells and/or transgene DNA; CTA313 median ~42 d	Recovery ~6 wk	Not systematically reported	Mostly G1–2 CRS; infection/cytopenia; non-peer-reviewed, overlapping updates
Allo CAR-T: UCAR-T19	Official conference (50)	dcSSc; N = 3; ≥3 mo	mRSS at reported follow-up	mRSS decreased in 3/3	Medication status inadequately reported	Peripheral blood CAR expansion in 2/3; undetectable ~14 d	Recovery 2–3 mo	No systematic tissue B-cell assessment	CRS 3/3, including G3 1/3; N = 3
Allo CAR-NK: QN-139b	Peer-reviewed case report (51)	dcSSc; N = 1; 6 mo	EUSTAR-AI, mRSS, ACR-CRISS, imaging/pathology at 6 mo	Clinical, imaging and pathology improvement in one patient	Complete TFR not documented	Peripheral blood CAR-NK cells; peak D7, near baseline D12	Recovery from M2; naive-dominant	Skin pathology and imaging; single patient	No CRS/ICANS/GVHD; G1 UTI; N = 1
Allo CAR-NK: XB-19.15/SNC-103	Peer-reviewed N = 3 plus conference expansion to N = 8 (52, 53)	SLE; N = 3/8; ~6–9 mo	SRI-4/DORIS and disease activity at reported visits	Preliminary disease-activity signals	2/3 stopped glucocorticoids in initial cohort; other therapy/duration unclear; not complete TFR	Peripheral blood transgene DNA by qPCR; below quantification by D3 initially	Recovery ~12 wk	Not systematically reported	No CRS initially; G1–2 CRS 2/8 in expansion; mixed source status
Allo CAR-NK: KN5501	Peer-reviewed case series (55)	SLE; N = 18; median ~11 mo	SLE activity, renal and serological outcomes through M6+	Disease activity and renal/serological signals	Medication reduced or stopped in some; complete TFR not a uniform endpoint	Peripheral blood CAR-NK cells; detected ≤14 d	Deep depletion 16/18; recovery 2–4 mo, naive-dominant	No standardized paired tissue B-cell assessment	G1 CRS 1/18; conditioning toxicity; uncontrolled series
Allo CAR-NK: cord blood CD19-BBz	Peer-reviewed phase 1 (54)	SLE; N = 5; 12–15 mo	SLEDAI-2K, SRI-4, LLDAS/DORIS and renal response at M6/M12	5/5 SRI-4 and LLDAS; 4/5 DORIS; 3/3 LN complete renal response at reported times	Non-glucocorticoid immunosuppressants stopped preconditioning and glucocorticoids tapered; complete TFR denominator/duration not reported	Peripheral blood CAR-NK cells by flow; undetectable by D21; molecular assay reported separately	Nadir ≤1 wk; recovery M1–3, naive-dominant	Reported tissue-related mechanistic analyses do not establish complete tissue depletion	G1 CRS 1/5; conditioning confounding; N = 5
*In vivo* LNP/linear mRNA: HN2301	Peer-reviewed correspondence (56)	Refractory SLE; N = 5; 3 mo	SLEDAI-2K through M3	SLEDAI-2K decreased in 5/5	Baseline glucocorticoids and hydroxychloroquine continued; complete TFR not assessed	Peripheral blood CAR mRNA and CAR+ CD8 cells; peak ~6 h, baseline by D2–3	Dose-dependent depletion ~7–10 d at 4 mg; reconstitution not characterized	No tissue/BM depletion evidence	Low-grade CRS 5/5; tocilizumab 3/5; N = 5, short follow-up
*In vivo* integrating lentiviral vector: JY231	Peer-reviewed phase 1 correspondence (41)	Progressive MS (7), MOGAD (3), gMG (3), IIM (3); N = 16; median 6 mo	Disease-specific clinical, imaging, serologic and CSF biomarker assessments	Preliminary within-participant clinical and biological improvements; heterogeneous endpoints and no control	Background-treatment withdrawal incompletely reported; complete TFR not established	PB VCN by ddPCR; median peak 53,962.5 copies/µg DNA at D11, sampled through M6; p24 undetectable by D4; VCN is not a viable-cell count	Rapid complete depletion; reappearance after >2 mo, naive-dominant	CAR/vector distribution assessed in CSF/BM; does not establish complete tissue B-cell depletion	Grade 1 CRS 11/16; no ICANS/LICATS; reversible grade 3/4 neutropenia 3/16; uncontrolled, short follow-up; no long-term genomic safety

AAV, ANCA-associated vasculitis; ACR-CRISS, American College of Rheumatology Combined Response Index in Systemic Sclerosis; BM, bone marrow; CRS, cytokine release syndrome; CSF, cerebrospinal fluid; dcSSc, diffuse cutaneous systemic sclerosis; ddPCR, droplet digital polymerase chain reaction; DORIS, Definitions of Remission in SLE; FU, follow-up; gMG, generalized myasthenia gravis; IIM, idiopathic inflammatory myopathy; LICATS, lentiviral *in vivo* CAR-T–associated toxicity syndrome; LLDAS, Lupus Low Disease Activity State; LN, lupus nephritis; MOGAD, myelin oligodendrocyte glycoprotein antibody-associated disease; mRSS, modified Rodnan skin score; MS, multiple sclerosis; NR, not reported; PB, peripheral blood; PD, pharmacodynamics; SLE, systemic lupus erythematosus; SRI-4, SLE Responder Index-4; SRP-IMNM, signal recognition particle immune-mediated necrotizing myopathy; TFR, treatment-free remission; VCN, vector copy number. Values are descriptive; heterogeneity precludes indirect efficacy comparisons. “Not reported” is not equivalent to absence.

Cross-platform response rates are not comparable. SLE studies use SRI-4, SLEDAI-2K, Lupus Low Disease Activity State (LLDAS), Definitions of Remission in SLE (DORIS), and renal endpoints; systemic sclerosis studies use modified Rodnan skin score (mRSS), ACR-CRISS, EUSTAR activity, imaging, or pathology; myositis and vasculitis use different outcomes. Organ involvement, prior treatment, conditioning, concomitant therapy, dose, repeat dosing, and assessment time also differ. We therefore report n/N and time points where available and avoid a common response metric.

### 4.2 Disease-specific and organ outcomes

Peer-reviewed allogeneic CAR-T reports describe short-term improvements in selected patients with SLE/lupus nephritis, systemic sclerosis, and immune-mediated necrotizing myopathy. TyU19 cohorts reported reductions in disease activity, muscle enzymes, skin scores, proteinuria, and autoantibodies over 6–12 months (29, 30, 44). Five YTS109 recipients with SLE/lupus nephritis achieved SRI-4 at month 3; four had major disease-activity reduction and one mild flare occurred by month 6 (45). Conference updates on FT819 and CTA311/CTA313 add treated patients but remain preliminary and may overlap (31–34, 46–49). In three SPPL3-null UCAR-T19 recipients with diffuse systemic sclerosis, mRSS improved, but the cohort cannot define response probability (50).

Allogeneic CAR-NK data support proof of concept, not equivalence. QN-139b was associated with clinical, imaging, and histopathological improvement in one patient with diffuse systemic sclerosis (51). XB-19.15/SNC-103 was reported in a three-patient peer-reviewed SLE cohort with conference expansion to eight patients (52, 53). Cord blood-derived CD19-BBz-NK was studied in five patients with refractory SLE (54). The largest peer-reviewed series, 18 KN5501-treated patients with SLE, reported disease-activity, renal, serological, and medication-reduction signals after conditioning and three infusions (55). Multicenter confirmation with prespecified endpoints is lacking.

HN2301 provides evidence that CD8-targeted LNP/linear mRNA can transiently generate circulating CD19 CAR-T cells *in vivo*, reduce peripheral B cells, and coincide with lower SLEDAI-2K scores through month 3 in five patients (56). Baseline glucocorticoids and hydroxychloroquine continued. The study does not establish sustained remission, B-cell reconstitution, long-term safety, lower cost, or generalizability to other *in vivo* systems.

JY231 provides clinical evidence for an integrating lentiviral *in vivo* CD19 CAR-T platform. Sixteen adults with neurologic IMID received a single intravenous dose of 2.0×10^9^ transducing units without lymphodepleting chemotherapy. At a median follow-up of 6 months, disease-specific clinical, imaging, serologic, and cerebrospinal-fluid biomarker improvements were reported. Endpoints were heterogeneous, however, and the uncontrolled study cannot estimate comparative efficacy. Reported CAR or vector signals in peripheral blood, cerebrospinal fluid, and bone marrow support delivery and target engagement, not complete tissue B-cell clearance or durable functional persistence (41).

Imaging and tissue findings are sparse. Selected reports describe changes in muscle MRI/biopsy, skin histology/ultrasonography, renal histology or spatial profiling, lung computed tomography, cardiac MRI, and brain PET-MR (29, 41, 44, 45, 51). Most derive from individual patients or very small cohorts without blinded central review or standardized acquisition. They support biological plausibility but not cross-platform comparison.

### 4.3 Medication reduction and treatment-free remission

Sustained complete treatment-free remission remains sparsely documented. Medication-free remission has been reported in selected allogeneic CAR-T recipients (30), but many reports describe glucocorticoid cessation, partial medication reduction, or incompletely characterized withdrawal rather than a uniform treatment-free endpoint (29, 30, 44, 46, 47, 49, 52, 54, 55, 57). HN2301 recipients continued baseline glucocorticoids and hydroxychloroquine (56), whereas background-treatment withdrawal was insufficiently reported for JY231 (41). Complete treatment-free remission requires documented disease-specific remission without systemic glucocorticoids, immunosuppressants, biologics, or other disease-modifying therapy; antimalarial use and duration should be reported separately.

Follow-up generally spans several months to approximately one year. Total follow-up should be distinguished from the duration of confirmed treatment-free remission and the observation period after B-cell reconstitution. These intervals are often short or incompletely reported, so late-relapse risk after B-cell recovery cannot be estimated reliably. Reconstitution itself neither proves restored tolerance nor implies inevitable relapse; absence of relapse over a short interval cannot establish durable control.

Attribution is also confounded by conditioning chemotherapy and concomitant glucocorticoids, hydroxychloroquine, or other immunosuppressants. Small uncontrolled cohorts cannot separate these effects from CAR-mediated activity. Future studies should prespecify remission and withdrawal criteria, record when both are satisfied, and report B-cell recovery, relapse, treatment re-initiation, and evaluable denominators at defined time points. Claims of durability should remain tied to explicitly documented observation intervals.

## 5 CAR kinetics, B-cell pharmacodynamics, and immune remodeling

### 5.1 Analyte-specific CAR kinetics

Three complementary but non-interchangeable readouts should be distinguished. Viable CAR-positive cells require cell-based assays with viability and lineage gating; CAR positivity alone does not establish effector function. Transgene DNA measured by qPCR or ddPCR represents molecular copy abundance, with interpretation dependent on the denominator and sample composition. CAR RNA may represent delivered RNA or cellular transcription, depending on platform, and does not independently establish surface expression or function. Molecular detectability, viable-cell persistence, functional activity, and clinical durability are separate outcomes.

For HN2301, CAR mRNA and CAR-positive CD8 T cells peaked at approximately 6 hours and approached baseline by days 2-3 (56). For JY231, peripheral-blood vector copy number (VCN) peaked at a median 53,962.5 copies/µg DNA on day 11 and was sampled through month 6; circulating p24 became undetectable by day 4 (41). DNA-based VCN is not a direct count of viable or functional CAR-positive cells, and its sampling window does not prove continuous functional persistence. Likewise, p24 clearance does not establish clearance of the transgene or engineered cells.

Negative results depend on sampling time, compartment, sample input and handling, and assay-specific limits of detection and quantification (LOD/LOQ). A signal may be detectable below the LOQ without being reliably quantifiable; “below LOQ” should not be reclassified as “undetectable.” An undetectable result means only that the assay criterion was not met in the tested sample at that time. **Figure 3**; **Supplementary Table S1** therefore separate analyte, specimen, assay, threshold, and observation window. Longer detectability is not a validated surrogate for deeper remodeling or remission and may prolong B-cell aplasia, hypogammaglobulinemia, or infection risk.

**Figure 3:**
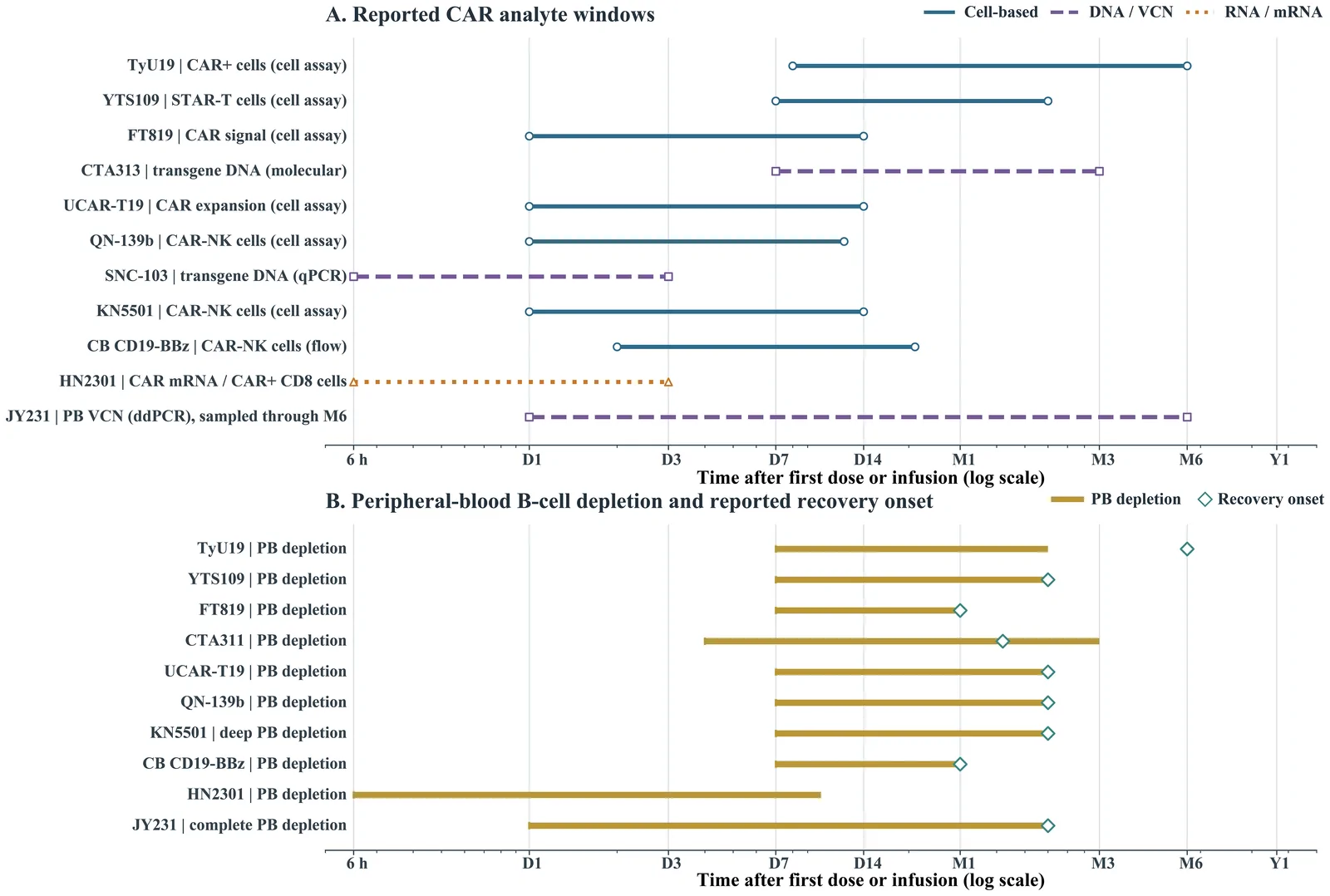
Analyte-specific CAR kinetics and peripheral B-cell pharmacodynamics. **(A)** Source-reported observation windows for CAR-positive cells, transgene/vector DNA, and CAR RNA, displayed separately by analyte. Cell viability is inferred only where supported by the assay. The JY231 VCN sampling window through month 6 does not establish continuous functional CAR-T-cell persistence. **(B)** Peripheral-blood B-cell depletion and reported recovery onset. Connecting lines summarize reported windows and do not establish continuous positivity or clearance at their endpoints. Specimens, assays, schedules, and thresholds differ. Below-LOQ results are distinguished from undetectable results; neither establishes complete cellular clearance. Peripheral-blood depletion does not establish tissue depletion. LOD, limit of detection; LOQ, limit of quantification; PB, peripheral blood; VCN, vector copy number.

### 5.2 Peripheral blood depletion, recovery, and tissue limits

All three clinically reported platform families can reduce circulating B-cell counts, but depth and duration vary with product, conditioning, dose, assay, and schedule. Several allogeneic CAR-T cohorts reported near-complete peripheral depletion within days to two weeks and recovery after approximately six weeks to several months (29, 30, 34, 44–50, 58). CAR-NK cohorts also reported substantial peripheral depletion despite shorter CAR detectability (51, 52, 54, 55, 57).In the 18-patient KN5501 series, 16/18 met the study threshold for deep depletion and recovery generally began within 2–4 months (55). For *in vivo* CAR-T, HN2301 produced dose-dependent peripheral depletion lasting approximately 7–10 days in the 4-mg group; subsequent reconstitution was not characterized (56). JY231 produced rapid complete peripheral depletion, with B-cell reappearance reported more than two months after infusion and a naive-dominant phenotype (41). These are peripheral pharmacodynamic observations. Blood depletion does not establish clearance of lymphoid-organ or inflamed-tissue B cells, bone-marrow precursors, or long-lived plasma cells. JY231-related CAR or vector signals in cerebrospinal fluid and bone marrow document sampled biodistribution, not complete tissue B-cell depletion (41). Paired blood–tissue sampling, where ethically justified, is needed to distinguish redistribution from compartmental depletion. **Figure 3**; **Supplementary Table S1** label tissue evidence separately.

### 5.3 Numerical reconstitution, functional recovery, and immune reset

Naive-dominant B-cell recovery, contraction of memory B-cell or plasmablast compartments, greater repertoire diversity, and loss of expanded clones have been described after selected allogeneic CAR-T and CAR-NK interventions. These findings indicate numerical and phenotypic reconstitution. Functional recovery additionally requires evidence of competent protective responses, such as preserved or restored vaccine-specific immunity and adequate immunoglobulin function without disproportionate infection.

An “immune reset” is a stronger, longitudinal hypothesis (59). It should require sustained disease-specific, complete treatment-free remission after target-cell recovery; durable suppression or loss of pathogenic autoreactive clones and immune programs; absence of clinical relapse; and preservation or restoration of protective immunity. Antigen-specific tolerance would require evidence that pathogenic self-reactivity is selectively controlled despite broader immune competence. In the JY231 report, replacement of expanded B-cell-receptor clonotypes was shown in one participant and is hypothesis-generating rather than proof of immune reset (41). Current naive-dominant recovery, serological improvement, or treatment reduction meets neither threshold alone. **Figure 4** therefore displays evidence of availability and source type, not ordinal maturity or platform performance.

**Figure 4:**
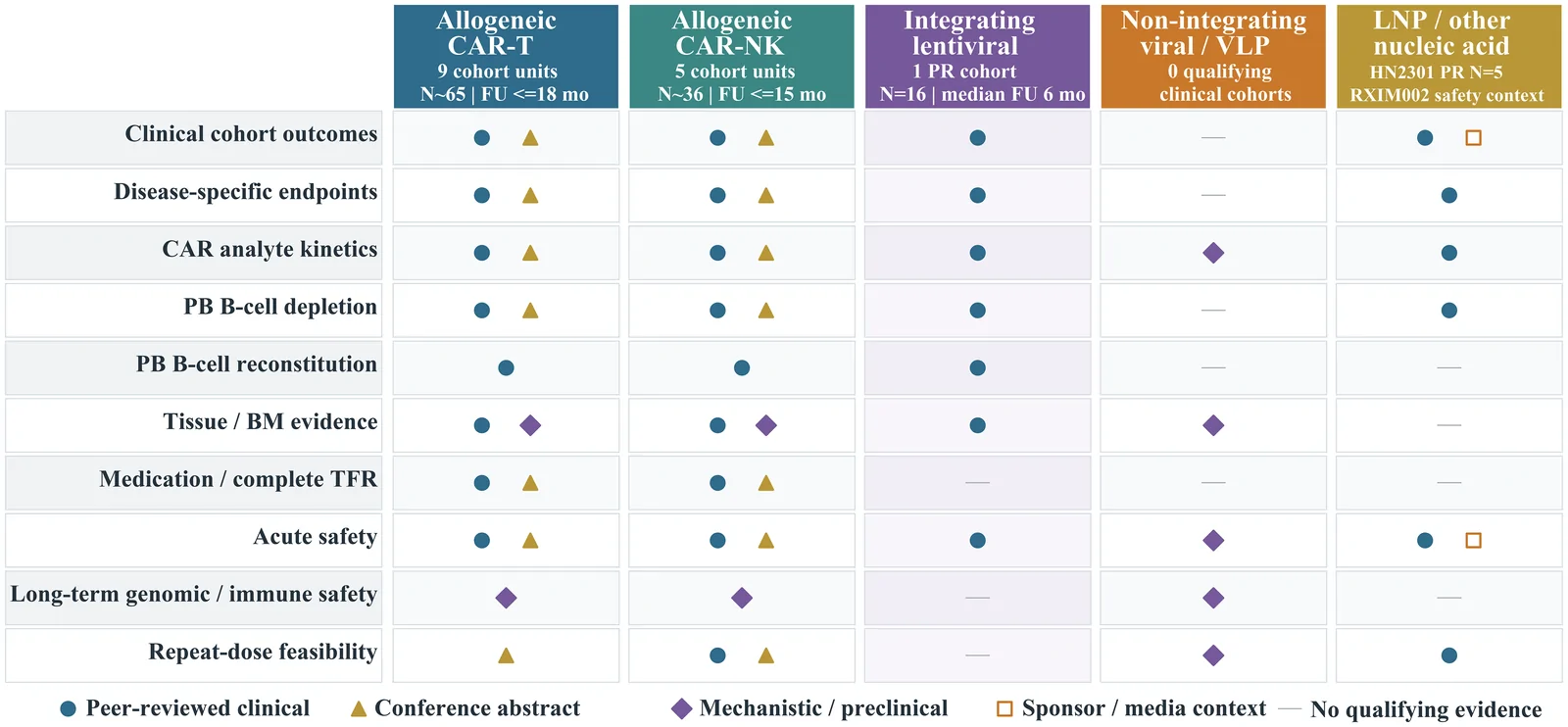
Evidence availability by domain and source type. This descriptive, non-ordinal matrix shows whether evidence is available and identifies source type. Symbols show that a source exists, not study quality, efficacy, or comparative maturity. The integrating-lentiviral column includes one peer-reviewed JY231 cohort (N = 16; median follow-up 6 months), while long-term genomic and immune safety remains unestablished. Counts are approximate because conference cohorts evolve and may overlap. Peripheral-blood pharmacodynamics do not establish tissue depletion. Counts are expressed as cohort units defined in Section 2.2. CA, conference abstract; circRNA, circular RNA; LNP, lipid nanoparticle; PR, peer-reviewed report; SR, sponsor/media report; VLP, virus-like particle.

## 6 Treatment delivery and safety

### 6.1 Conditioning and dosing

Most allogeneic CAR-T studies used fludarabine/cyclophosphamide or another reduced-intensity regimen, although FT819 was also evaluated with fludarabine-free or no conditioning (32). Conditioning can facilitate expansion but contributes to cytopenias, infection, and early immune perturbation. CAR-NK regimens are heterogeneous and commonly involve repeated infusions; KN5501 recipients received fludarabine 25 mg/m²/day plus cyclophosphamide 300 mg/m²/day on days −5 to −3 (55). HN2301 used repeated LNP/mRNA doses without conventional lymphodepletion while baseline glucocorticoids and hydroxychloroquine continued (56). JY231 was administered once at 2.0×10^9^ transducing units without conventional lymphodepleting chemotherapy (41). These regimens are different cumulative exposures.

### 6.2 Acute safety and attribution

Relevant events include cytokine release syndrome (CRS), immune effector cell-associated neurotoxicity syndrome (ICANS), infusion reactions, GVHD for donor T-cell products, conditioning-related cytopenias and infection, and depletion-related hypogammaglobulinemia or loss of vaccine immunity. Prior immunosuppression, active disease, conditioning, and CAR activity complicate attribution. Small samples cannot establish a low incidence of uncommon or delayed events.

All three reported UCAR-T19 recipients developed CRS, including one grade 3 event (50). Infections, cytopenias, and immunoglobulin reductions have occurred in several allogeneic CAR-T studies (29, 45–50). CAR-NK cohorts mainly reported low-grade CRS, infection, or conditioning-related events (51, 55, 57). One preliminary CNTY-101 recipient experienced catheter-associated bacteremia, which resolved (57). All five HN2301 recipients had low-grade CRS; three received tocilizumab, and transient cytopenias and mild infections were reported (56). In the 16-participant JY231 report, 11 had grade 1 CRS, no ICANS or lentiviral *in vivo* CAR-T–associated toxicity syndrome was reported, and three had reversible grade 3 or 4 neutropenia (41).

Within the search period, RiboX Therapeutics reported a death occurring after RXIM002 treatment in a participant with systemic sclerosis and significant pre-existing conditions (60). A contemporaneous news report supplied additional context (61). Neither source provides independent adjudication or sufficient clinical detail to infer whether the death was related to the disease, concomitant factors, the circular RNA cargo, the LNP, CAR activity, or another mechanism. RXIM002 was therefore not included in efficacy counts, and the report is presented only as an unadjudicated safety signal requiring transparent investigation.

### 6.3 Delayed and platform-specific risks

Edited allogeneic products require assessment of nuclease off-target changes, on-target large rearrangements, vector copy number and integration sites, chromosomal abnormalities, and clonal evolution. Surveillance should include delayed GVHD, rejection, marrow abnormalities, malignancy, infection, and persistent immune dysfunction (25, 26, 28, 36, 62–64).

*In vivo* risks depend on vector and cargo. Integrating vectors require insertion-site, genotoxicity, persistence, shedding, and unintended-transduction assessment. JY231 integration-site analysis was polyclonal and predominantly intronic, without reported oncogene-related clonal expansion during the short observation period; these findings do not establish long-term genomic safety (41). Non-integrating viral or virus-like systems may still cause off-target tropism, shedding, immune responses, and neutralizing antibodies that limit redosing. LNP/RNA systems reduce intended integration but require cell- and organ-specific biodistribution, inflammatory response, cargo/carrier persistence, anti-carrier immunity, and repeat-exposure testing (13, 38–40, 42, 43, 65, 66). Transient expression does not by itself establish safe redosing.

Long-term protocols should prespecify product-specific monitoring: (1) integration sites, VCN, and insertion distribution for integrating systems; (2) clonal abundance, diversity, persistence, genomic integrity, and, for edited products, risk-informed off-target and unintended on-target changes; (3) onset, organ involvement, severity, treatment, and resolution of late GVHD; (4) serial immunoglobulins, B-cell counts and subsets, hypogammaglobulinemia, and replacement therapy; (5) infection timing, pathogen, severity, recurrence, viral reactivation, prophylaxis, and outcomes; and (6) blood counts plus evaluation of secondary malignancies or marrow abnormalities, including lineage/origin and CAR/vector sequences where feasible. Baseline assessments, sampling intervals, assay sensitivity, follow-up duration, and triggers for further investigation should be defined. Persistent clonal dominance warrants investigation, but clonal expansion alone does not establish malignant transformation (67–71).

## 7 Manufacturing, regulation, and access

Allogeneic products may replace patient-specific manufacture with donor or renewable banks, batch production, cryopreservation, and inventory (27). Performance still depends on source variability, editing consistency, expansion-related drift, cryorecovery, potency, batch comparability, and contamination or failure management. CAR-NK adds source- and cytokine-dependent expansion and multidose supply (72). *In vivo* engineering removes ex vivo cell manipulation but shifts the bottleneck to GMP vector or nanoparticle manufacture, particle composition and stability, cell-specific delivery, biodistribution, and lot reproducibility (40, 73).

Regulatory classification follows the administered product, jurisdiction, and mechanism; it should not be reduced to a single universal label. In the United States, an administered vector or nucleic acid designed to genetically modify cells *in vivo* is generally evaluated within the human gene-therapy framework, with modality-specific expectations for chemistry, manufacturing and controls, biodistribution, shedding, unintended transduction, genomic consequences, and long-term follow-up (67). In the European Union, ex vivo manipulated cells may meet advanced therapy medicinal product definitions, while *in vivo* vector or nucleic-acid products are assessed under applicable gene-therapy and genetically modified organism frameworks (74, 75). The administered vector/LNP product must be distinguished from the CAR-positive cells generated inside the patient; both inform pharmacology and risk, but they are not interchangeable regulatory articles.

Lower cost and broader access remain hypotheses. Total cost includes manufacturing, conditioning, hospitalization, monitoring, toxicity management, repeat dosing, immune surveillance, and long-term follow-up. Community delivery would require predictable toxicity, simplified conditioning, validated referral and emergency pathways, reimbursement, and implementation evidence. Comparative cost, time-to-treatment, budget-impact, and real-world access studies are largely absent (12, 14, 15, 27, 76).

Anti-CD20 antibodies and T-cell engagers are active comparators (1, 8, 77). Anti-CD20 therapy offers established dosing and access but may incompletely deplete tissue B cells and CD20-negative plasmablasts or plasma cells, with retreatment and immunoglobulin or infection monitoring often needed (78). CD19×CD3 engagers are immediately available, dose-titratable, and interruptible (79, 80); but IMID evidence remains early and T-cell dependence, repeat dosing, CRS or neurologic toxicity, infection, and hypogammaglobulinemia require study (81–83). BCMA×CD3 engagers have less IMID evidence than oncology evidence (8, 77). Allogeneic and *in vivo* CAR platforms therefore require a demonstrated advantage—deeper or more durable depletion, activity after comparator failure, treatment-free control, or lower cumulative burden—while accounting for conditioning, alloreactivity, biodistribution, genomic, and repeat-dosing risks.

## 8 Biological boundaries across IMIDs

The strongest rationale for CD19- or BCMA-directed CAR therapy is in diseases where B cells, plasmablasts, plasma cells, autoantibodies, or B-cell antigen presentation materially sustain disease (1, 77). Even within SLE, systemic sclerosis, myositis, and vasculitis, target dependence varies by phenotype, duration, tissue compartment, and established damage. Fibroblast activation, endothelial injury, tissue-resident T cells, macrophages, and self-sustaining fibrosis can persist despite peripheral depletion (7, 84, 85).

The rationale is weaker for predominantly TNF-, IL-17/IL-23-, type 2 cytokine-, inflammasome-, innate immune-, stromal-, or T-cell-driven IMIDs without a demonstrated pathogenic B-cell compartment (9). Applying CD19 depletion in such settings may add conditioning and immune toxicity without addressing the dominant mechanism (86). Future strategies targeting autoreactive T cells, myeloid cells, fibroblasts, or regulatory pathways require independent disease-specific validation (87–89).

## 9 Critical appraisal and translational priorities

Current evidence establishes feasibility, not comparative superiority. Limitations include small uncontrolled cohorts, short and unequal follow-up, concentration in SLE, heterogeneous conditioning and background therapy, non-comparable assays and endpoints, inconsistent medication reporting, cohort overlap, incomplete adverse-event reporting, and reliance on preliminary or sponsor sources. Publication and selective-reporting bias are likely.

Future studies should use anti-CD20 therapy and appropriate T-cell engagers as prespecified active comparators (8, 77, 90), with endpoints covering time to treatment, cumulative exposure, blood and tissue pharmacodynamics, B-cell and plasma-cell subsets, treatment-free remission, relapse, immunoglobulins, infections, CRS or ICANS, and patient burden. Early response, molecular detectability, or peripheral B-cell depletion alone should not establish superiority; until comparative benefit is shown, these newer CAR platforms remain hypothesis-driven alternatives.

Prospective multicenter studies should enroll defined disease- and product-specific cohorts. Early phases should establish safety, dose, and feasibility, followed where feasible by randomized studies with active comparators. Protocols should prespecify disease-specific outcomes, medication tapering and rescue rules, relapse, sustained complete treatment-free remission, organ damage, patient-reported outcomes, and adverse-event attribution. Conditioning and concomitant treatment should be standardized where possible and documented longitudinally, with follow-up extending beyond B-cell reconstitution.

Biomarker programs should distinguish viable CAR-positive cells, transgene DNA, and CAR RNA using harmonized specimen handling, sampling windows, units, and assay-specific LOD/LOQ. Longitudinal analyses should relate B-cell subsets, autoreactive clones or immune programs, immunoglobulins, vaccine-specific responses, infections, and ethically justified tissue findings to clinical outcomes. Immune reset remains an exploratory hypothesis requiring convergent evidence of sustained complete treatment-free remission after target-cell recovery, durable control of pathogenic self-reactivity, and preserved protective immunity.

Regulatory planning should include early product-specific agency dialog on potency, manufacturing comparability, biodistribution, genomic risk, and long-term surveillance. Time to treatment, total care costs, center requirements, reimbursement barriers, and representation across populations should be measured prospectively. No platform yet has an established clinical niche or comparative advantage. These approaches should remain investigational until adequately powered studies and long-term registries show reproducible benefit, acceptable acute and delayed risk, scalable manufacture, and equitable implementation.

## Supplementary material

Supplementary Table 1 Cohort-level clinical evidence with disease-specific endpoints, medication status, analyte/assay fields, peripheral blood pharmacodynamics, and tissue evidence.

Supplementary Table 2 Preclinical and clinical platform inventories for allogeneic CAR-T, allogeneic CAR-NK, and in vivo CAR-T.

Supplementary Table 3 Preclinical and clinical platform inventories for allogeneic CAR-T, allogeneic CAR-NK, and in vivo CAR-T.

Supplementary Table 4 Preclinical and clinical platform inventories for allogeneic CAR-T, allogeneic CAR-NK, and in vivo CAR-T.

Supplementary Table 5 Registered studies of allogeneic CAR-T, allogeneic CAR-NK, and in vivo CAR-T; registry status is dynamic and should be rechecked before submission.

Supplementary Table 6 Registered studies of allogeneic CAR-T, allogeneic CAR-NK, and in vivo CAR-T; registry status is dynamic and should be rechecked before submission.

Supplementary Table 7 Registered studies of allogeneic CAR-T, allogeneic CAR-NK, and in vivo CAR-T; registry status is dynamic and should be rechecked before submission.
